# Evolutionary aspects of self- and world consciousness in vertebrates

**DOI:** 10.3389/fnhum.2015.00157

**Published:** 2015-03-26

**Authors:** Franco Fabbro, Salvatore M. Aglioti, Massimo Bergamasco, Andrea Clarici, Jaak Panksepp

**Affiliations:** ^1^Department of Human Sciences, University of UdineUdine, Italy; ^2^Perceptual Robotics Laboratory, Scuola Superiore Sant'AnnaPisa, Italy; ^3^Department of Psychology, Sapienza University of RomeRome, Italy; ^4^Fondazione Santa Lucia, IRCCSRome, Italy; ^5^Psychiatric Unit, Department of Medical, Surgical and Health Sciences, University of TriesteTrieste, Italy; ^6^Department of Veterinary and Comparative Anatomy, Pharmacology, and Physiology, College of Veterinary Medicine, Washington State UniversityPullman, WA, USA

**Keywords:** nervous system evolution, vertebrate brain, self and world awareness, mental time traveling, theory of mind

## Abstract

Although most aspects of world and self-consciousness are inherently subjective, neuroscience studies in humans and non-human animals provide correlational and causative indices of specific links between brain activity and representation of the self and the world. In this article we review neuroanatomic, neurophysiological and neuropsychological data supporting the hypothesis that different levels of self and world representation in vertebrates rely upon (i) a “basal” subcortical system that includes brainstem, hypothalamus and central thalamic nuclei and that may underpin the primary (or anoetic) consciousness likely present in all vertebrates; and (ii) a forebrain system that include the medial and lateral structures of the cerebral hemispheres and may sustain the most sophisticated forms of consciousness [e.g., noetic (knowledge based) and autonoetic, reflective knowledge]. We posit a mutual, bidirectional functional influence between these two major brain circuits. We conclude that basic aspects of consciousness like primary self and core self (based on anoetic and noetic consciousness) are present in many species of vertebrates and that, even self-consciousness (autonoetic consciousness) does not seem to be a prerogative of humans and of some non-human primates but may, to a certain extent, be present in some other mammals and birds

*Look in the mirror, and don't be tempted to equate transient domination with either intrinsic superiority or prospects for extended survival*.

*Stephen Jay (Gould, 2011)*

## Introduction

Under physiological conditions the experience of returning from the inner world (as in dreams) to the outer world as a reflectively conscious, sentient being, with self-awareness, occurs very often (e.g., every time one wakes up). However, understanding the link between consciousness and representation of the self existentially in both social and non-social worlds, is a challenging enterprise for both psychology and neuroscience. Consciousness can be considered as the appearance of a world during both waking or dreaming states (Edelman, 2004; Revonsuo, 2006, 2010, Metzinger, 2009). It is often divided into primary (anoetic) consciousness mainly related to perception-, affect-, and action- related representations; and in higher- order consciousness linked to interpretation of the primary consciousness contents (noetic) including self-related notions (autonoetic) of past and future (Edelman, 1989; Seth et al., 2005; Kahneman, 2011).

In addition to the difficulties linked to the notion of consciousness as a “hard problem,” i.e., why we have phenomenal experiences (Chalmers, 1995), several other foundational issues should be taken into account. The first is that any approach to the scientific investigation of this question needs to integrate data from first (subjective) and third-person (objective) perspectives. The second has to do with the need to deal with philosophical reflections, with abundant historical “baggage,” related to the different levels of analysis of the issues under investigation. The third is the problem of making explicit the philosophical positions of scientific investigators of consciousness, since multiple levels of brain-mind control need to be considered. Such levels range from anoetic (experience without knowledge) and noetic (experience with knowledge), to autonoetic consciousness which consists of “time travel” where the minds eye can explore in a self-centered perspective past experiences and future hopes and aspirations (see Edelman, 2004).

Extending the analysis to the different classes of vertebrates is even more challenging because, not only is difficult for humans to understand or experience other human sentient beings, but it is even more complex to imagine “what is it like to be a bat” (Nagel, 1974). Moreover, knowledge about the relationship between the mind of animals and the anatomical and physiological organization of their nervous system is meager. Taxonomically, a large part of the phylum chordata is constituted by the subphylum vertebrata that includes a variety of species (about 4% of all described animal species) ranging from fish, amphibians, reptiles and birds to mammals. All the different vertebrate species are endowed with a common brain archetype divided into telencephalon and diencephalon (collectively referred to as forebrain), mesencephalon (midbrain) and rhombencephalon (hindbrain). Given the widely held causal relation between brain and behavior, an important question for psychology, neuroscience and even philosophy is whether and to what extent all the different vertebrate species share the mechanisms involved in the evolutionary emergence of phenomenal experiences (including affective, interoceptive, and exteroceptive varieties) to the much higher ability to mentally represent the self as an actor in the world. In the following we examine this extant issue by starting from humans in whom a variety of simple and complex forms of consciousness exist.

Knowledge about the meaning of world and self-representation deriving from phenomenological, neuropsychological and neurophysiological approaches will be reviewed. Since phenomenological (subjective) evidence can be obtained only in humans (but only semi-directly through language), we must tackle evolutionarily related issues in the other classes of vertebrates (fishes, amphibians, reptiles, birds, and mammals) by reviewing not only neuroanatomical, neuropsychological and neurophysiological data. Moreover behavioral data hinting at the presence of affective experiences will be discussed by evaluating the rewarding and punishing properties, as inferred from deep brain stimulation (DBS) of brain emotional circuits. It is important to note that we do not intend to use the phylogenetic taxonomy for establishing a sort of hierarchy among vertebrates and we do not intend to attribute to, say, living reptiles a higher or lower place with respect to mammals. We simply mean that the different classes of vertebrates represent different genealogical lines originating from a common origin and that each class can be described by referring to the complexity of their nervous system (Butler and Hodos, 1996; Denton, 2006), and to functional homologies of the brain structures involved in primary-process emotions (Panksepp, 1998a, 2011). In the following, we discuss homologies, analogies and differences in the way self and world representation manifest in the different vertebrate species.

## Consciousness in human beings

Neuroscience studies indicate that the human brain continuously creates the conscious experience of being in the world (i.e., “I am in this outside-me place, now”; Revonsuo, 2006, 2010; Metzinger, 2009). Consciousness emerges from the brain's ability to construct a complex representation of the world and the self (Edelman, 2004). That both the self and the world are constructed by the brain is strongly suggested by the profound changes of self and world consciousness following cerebral lesions and during dreaming, a condition where external inputs are blocked (Frith, 2007). It is worth noting that lesions of specific brain regions may bring about domain-specific deficits of awareness. Lesions to different visual areas, for example, may induce defects in the recognition the form (apperceptive agnosia) or the movement of an object (akinetopsia, Zeki, 2009) and lesions to higher-order cortical regions may induce defective awareness of space (e.g., hemispatial neglect, Kourtzi and Connor, 2011). Complex domain-specific deficits following frontal or occipito-parietal regions such as anosoagnosia for hemiplegia (Moro et al., 2011) or for visual deficits (Anton Babinsky's syndrome) have also been reported (Feinberg and Keenan, 2005). However, we are not aware that our experience of the world and of the self are brain constructions and that self and world represent models through which information is processed. In philosophical parlance, the illusion that such models are the reality is called *transparency* (Revonsuo, 2006; Metzinger, 2009). It is worth noting that the brain's construction of the world and self is not arbitrary but it takes into account the operational interactions learned at both phylogenetic and ontogenetic levels (Jerison, 1973; Striedter, 2005). More specifically, the world representations created by the brain are highly adaptive and allow individuals to implement a number of effective operations like moving in the world, maintaining bodily and affective homeostasis, developing plans for self- fulfillment (Geary, 2007). Importantly, however, several examples speak in favor of the inherent link between “real” and “mentally” created worlds, or as it has been also called “the objectively perceived reality” vs. “the subjectively conceived reality” (Lakoff and Johnson, 1980; Humphrey, 1992, 2011). Conscious awareness is a simulation *per se* and does not put the subject in direct contact with reality. It is thus possible to think of mental experiments in which even an expert pilot unaware to be in a very realistic flight simulator believes he is operating on a real airplane (Metzinger, 2009). Moreover, representations concerning the world and the self may often be contradictory, as indicated by the fact that illusions may fool vision but not action suggesting errors may occur in allocentric (i.e., world-based) visual coordinates but not in ego-centric (i.e., self-based) motor coordinates. For example, people are susceptible to the “Titchener circles” illusion in which the same target circle appears to be larger when is surrounded by a circular array of smaller than larger circles. Crucially, the same participants who are fooled by the illusion when making perceptual judgments, when asked to pick up the central circle scale their grip aperture on the basis of the true size of the target disc and not of its illusory size (Aglioti et al., 1995). In a complementary vein, illusory doubling of tactile stimuli may occur only if a conflict occurs between egocentric (body-based) and allocentric (world centered) coordinate frames like for example in the Aristotle's diploaesthesia. Indeed, one object touching the contact area between the crossed index and middle fingers is perceived as two objects localized on the lateral surface of each finger (Bufalari et al., 2014). Striking contradictions occur also during dreaming (Hobson, 1989, 1999), suggesting an analogy between this state of consciousness and the above illusions. All these states may be of fundamental importance for understanding the problem of consciousness (Metzinger, 2009; Revonsuo, 2010).

Human consciousness can be analyzed according to different levels, the most significant of which are the phenomenological, the neurological and the neurophysiological ones (Revonsuo, 2006). Each level possesses specific properties and rules. Importantly, the lower levels influence the higher levels and vice-versa (Revonsuo, 2006; Graziano and Kastner, 2011; Graziano, 2013), reflecting the bottom-up ontogenetic/epigenetic development of mind. We acknowledge, however, that different epistemological perspectives suggest different models ranging from hierarchical (Craver and Darden, 2001; Neisser, 2012), to relatively independent ones (Ayala and Arp, 2010).

### Phenomenological level

In addition to the classical third-person-perspective analysis (3PP: a perspective in which the whole universe is given as existent independently from the observer) it is necessary to envisage a first person perspective (1PP: in this perspective the objects in space are necessarily related to an agent) (Revonsuo, 2006, 2010). At this level, consciousness is an immediate, undeniable fact of experience, i.e., a “world-for-me” and, more in general “a-world-for-someone,” which comes in two general types—interoceptive (from body) and even more within brain experiences (e.g., homeostatic states such as hunger and thirst and affective/emotional/mood states) and the more explicit exteroceptively driven sensory-driven experiences (e.g., perceptions). Since the phenomenal world is perceived as “present-for-me,” it can be considered as composed by a phenomenal space and by a self, which is the basis for building an egocentric frame of reference. The ego-center is single and metaphorically located behind the bridge of the nose, inside our head (Merker, 2007), but more realistically arising from certain within-brain experiences. The objects belonging to the world are located in the phenomenal dimension characterized by spatial and temporal attributes (Revonsuo, 2006).

The phenomenal self, as pointed out by William James, possesses the characteristics of being “partially known and partially knower, partly object and partly subject” (James, 1892/1948, p. 176). Already at the phenomenological level, the self does not seem to be an object, rather a within-brain process, a continuous stream of experiences and thoughts. The self seems to disappear during deep slow-wave sleep, and sometimes to dissolve or have less defined boundaries, also during dreaming (Revonsuo, 2005). Tellingly, the self reappears with greater perceptual depth and clarity when we wake up, as already noticed by the Heraclitus more than 25 centuries ago (Haxton and Hillman, 2001). The self seems to consist of at least two components, probably arranged in layers. The first, called the “observer” and corresponding to what James defined the “I” (James, 1890/1950), is only scarcely investigated by the western psychology that historically did not provide enough attention to introspection. An exception to this neglect may come from psychoanalysis that made serious attempts to distinguish between the Ego and the Self (Treurniet, 1979). The “I,” on the other hand, has been deeply considered in the Eastern world especially in the context of Buddhism (Horney, 1950; Tsering, 2006). The second component called the “observed” and corresponding what James defined the “Me,” refers to the content of the experience (e.g., thoughts, sensations, emotions, and perceived objects; Tagini and Raffone, 2010).

### Disorders of consciousness in humans

Human consciousness has been operationalized as mainly formed by two components, namely arousal (wakefulness or vigilance, with phenomenal contents) and awareness (which implies higher recognition of those contents). This latter component is further divided in external awareness concerning the sensorial analysis of the environment and the internal awareness (or self-consciousness) concerning the inner mental representation of the self (Demertzi et al., 2013a,b). While arousal is linked to activity of brain stem neural populations connected both directly or indirectly with the cerebral cortex, awareness depends on the functional integrity of cerebral cortex and thalamus. From an evolutionary perspective, the former is commonly assumed to be foundational for the latter.

Neuroimaging studies indicate that awareness depends on the integrity of a large cortical network with medial fronto-parietal structures dealing with processing of internal states mainly related to self-consciousness (mind wandering, inner speech, autobiographical memory recall) and lateral cortical structures mainly dealing with awareness of the external world (Vanhaudenhuyse et al., 2011; Giacino et al., 2014). Fundamental clues to the understanding of structures and mechanisms necessary and sufficient to the appearance of conscious self and world representation come from the analysis of patients in whom cerebral damage alters different forms of consciousness, like coma, vegetative state (VS), and minimally conscious state (MCS) (Laureys et al., 2004; Demertzi et al., 2013a,b; Owen, 2013).

Functional imaging studies indicate that medial and posterior cortical regions like the precuneus and the posterior cingulate cortex play a crucial role in human awareness. These regions, for example, do show high metabolic activity during aware wakefulness. By contrast they are deactivated in profound anesthesia, in VS patients and in severely demented patients. Precuneus and posterior cingulate cortex are also hypoactive in MSC (Ranganath and Ritchey, 2012; Mashour and Alkire, 2013a,b; Giacino et al., 2014). Relevant to this issue are the recent studies using transcranial magnetic stimulation (TMS) to perturb the cortex and EEG to record the effects of such perturbation. These studies demonstrate the importance of cortico-cortical and cortico-subcortical functional connectivity in human conscious awareness. More specifically the perturbation induced by TMS on EEG in VS patients indicate a severe defect of connectivity that is reminiscent of deep sleep and general anaesthesia states (Casali et al., 2013; Sarasso et al., 2014). MSC is instead characterized by complex EEG activity suggesting no alteration of connectivity. Such complex EEG activity, typically found in in healthy controls, is also observed in Locked-in patients who are aware of the self and of the environment in spite of their severe de-efferentation (Rosanova et al., 2012; Tononi, 2012). The notion that consciousness is related to an optimal functional connectivity is in keeping with influential theories of consciousness developed in psychology (e.g., the Global Workspace Theory hypothesis, Baars, 1988, 2002) and neurobiology (e.g., the Dynamic Core Theory, Edelman et al., 2011).

### Neuropsychological alterations of self consciousness

Empirical and philosophical studies suggest that the self, in human beings, is formed by diverse neuropsychological components (Stern, 1985; Gallagher, 2000; Northoff et al., 2006). Already (James, 1890/1950) in his Principles of Psychology (Chapter X, “The Consciousness of Self”) proposed distinctions between a physical self, a mental self, and a spiritual self. This distinction reappeared in the contemporary neuroscience debate. Particularly important are the studies of complex human pathologies, e.g., epilepsy, consciousness disorders and the clinical conditions that generate self-disorders (Feinberg and Keenan, 2005). Overall the following components of human self can be defined: (i) the proto-self; (ii) the core self; (iii) the self-consciousness; (iv) the narrative self.

- The “proto-self” (Damasio, 1999, 2010), can be defined as the most ancient form of coherent world and self-representation (Panksepp and Northoff, 2009) and it can be considered as a primary template that helps organisms to focus on the fundamental intrinsic aspects (e.g., visceral and motor) of the self. It can be considered as an organizational unit providing some kind of foundational coordination for all sensations (Humphrey, 1992, 2011) and primordial emotions (Panksepp, 1998a,b; Denton, 2006). Behavioral studies indicate that human newborns do exhibit a complex affective life as indexed by basic emotions as pain, joy, disgust, anger and by self-relevant acts like hand-mouth coordination movements (Stern, 1985; Rochat, 2013). For example, newborn babies may purposely move their hand to counteract external forces applied to their wrist with the clear aim to keep seeing their hand and thus exert a better control on their own action (Van der Meer and Lee, 1995). It is also relevant that human neonates exhibit a higher number of head turns toward tactile stimuli delivered on their cheek by an examiner (external stimulation) with respect to tactile stimuli delivered on the cheek by themselves (self-stimulation). This behavioral pattern speaks in favor of the innate ability to discriminate whether tactile stimuli are delivered by the self vs. others (Rochat and Hespos, 1997).
- “The “core self” is constituted by the pre-reflective consciousness of oneself as an immediate, embodied subject of experience (Damasio, 1999, 2010; Parvizi and Damasio, 2001). The core self involves a number of components, including the sense of ownership (i.e., the feeling the body is mine), and sense of agency (i.e., the feeling I am the one who caused my movement). These dimensions are pre-reflective, implicit, or tacit aspects of our experience (Vogeley and Gallagher, 2013, p. 119). The core self is connected to semantic memory systems and also to affective systems (Tulving, 2002a; Northoff and Panksepp, 2008; Panksepp and Northoff, 2009). Such basic forms of self and world representations likely operate only in the present moment (Edelman, 1989; Edelman and Tononi, 2000). Individuals who possess this form of consciousness show self-recognition and recognition of objects in the world (Tulving, 2002b). Many nonhuman animals, especially mammals and birds, for example, seem to be endowed with well-developed knowledge-of-the-world (semantic memory) and to flexibly utilize this information (Tulving, 2002a, p. 6). Conditioning studies indicate that semantic memory systems are present in birds. Pigeons for example, can learn to discriminate images of trees and categorize them according to whether they are with or without leaves (Herrnstein, 1979, 1990) as well as to form the “concept” of car, chair, human face (Cook and Smith, 2006). Not only mammals but also several species of fishes, reptiles and birds can encode the features of the environment in “map-like memory representation” (Bshary et al., 2002). Moreover, selective lesions of the teleost lateral pallium (a structure analogous to the mammalian hippocampus) in goldfish trained in a variety of spatial memory tasks induce conspicuous spatial memory deficits (Broglio et al., 2011). Two important characteristics of the core self are the sense of ownership (i.e., of owing a body) and the sense of agency (i.e., of being the actor of one's own actions).
- “*Self-consciousness*” has typically been explored in developmental and comparative psychology using mirror recognition tests that explore how humans or other animals react to surreptitiously introduced changes (e.g., a blot on their body) of their image reflected in a mirror. Only at the age of 18 months children become capable of self-recognizing themselves in the mirror and to understand that pictures represent other people. According to Perner (1991) mirror self-recognition implies to own the representation of the model of a real self as well as the model of the reflected-in-a-mirror model. The same test has been used in several vertebrate species. Although controversies have arisen about the validity of the mark test, studies suggest that chimpanzees (after the age of 28 month), orangutans, macaques, dolphins, elephants and corvid birds may show evidence of mirror self-exploration. By contrast, animals like dogs or cats either ignore the reflected image or react to the image as though they were in front of a rival individual (Devue and Brédart, 2011; Mashour and Alkire, 2013b). It is worth noting that the mirror test may not represent definitive evidence for the presence or absence of self-recognition and spurious variables (like scarce motivation to watch reflected images) may influence the test (Gallup et al., 2013).
- The narrative self (Gallagher, 2000; Boyd, 2009; Damasio, 2010) refers to the capability of handle episodic-type, declarative memories that unify the self into a coherent story. Prerequisites for developing a fully blown narrative self may include language (Gazzaniga, 2002, 2011; Baddeley et al., 2009) and the neuro-psychic device that allows individuals to mentally travel in time (Wheeler et al., 1997; Tulving, 2002a,b; Corballis, 2012). Such a device makes possible not only the aware recall of past events (i.e., autobiographical memory) but also to imagine possible future events including one's own death (Valentini et al., 2014). The ability to mental traveling in time seems to be underpinned by a variety of neural regions involved in memory (e.g., the hippocampus) and in the aware perception of the self (e.g., mid-line prefrontal and parietal regions, Schacter et al., 2007). The capability to mentally travel in time, an ability linked to the task-unrelated thoughts that characterize mind wandering, probably appeared more than one million years ago in the genus *Homo habilis* and is possibly at the basis of building lithic tools (Corballis, 2012; Fabbro and Crescentini, 2014). Importantly, the mind may wander not only in time by also in others' mind. Related to this is the development of shared intentionality (joint intentions and attention) and the ability to know (or the belief to be able to know) what other individuals think or believe, the so-called theory of mind that seems to be mainly a human feature (Tomasello et al., 2005; Corballis, 2012). It has been suggested that self-referential cognition, inner speech, mind wandering and autobiographical memory are important components of self-awareness a complex function likely underpinned by medial brain areas (Vanhaudenhuyse et al., 2011; Demertzi et al., 2013a,b; Campanella et al., 2014).

### Neurophysiological correlates of consciousness

One of the most significant discoveries in the contemporary neurophysiological research has been the correlation between neural activity of specific neuronal populations and conscious experience. It has been demonstrated that the conscious recognition of a visual stimulus correlates with synchronized discharge frequencies of neurons around 40 Hz (or in the gamma band: 20–80 Hz). In binocular rivalry experiments (Logothetis and Schall, 1989), for example, the gamma band is correlated with the activity of the visual areas that are involved in the processing of the dominant (conscious) stimulus. The same gamma band activity decreases in neurons that are involved in the processing of suppressed (non-conscious) stimuli (Engel and Singer, 2001; Fries, 2009). Moreover, neural activity synchronized around 40 Hz has been then correlated with the phenomenal content of visual awareness and has been associated to the efficiency of cortico-cortical connections and/or to specific and nonspecific thalamo-cortical loops (Llinás, 2001; Edelman, 2004). The gamma band specific neuronal activity seems associated to the “binding” of the disparate elements that compounds a unitary conscious percept. Interestingly, such activity has been found also at the subcortical level (e.g., the superior colliculus or the optic tectum) (Brecht et al., 2001; Merker, 2007).

Neurophysiology may help substantially to understand the link between states of consciousness and brain activity. Seminal studies indicate that it takes a long time (about 300–500 ms) to sensory stimuli to reach the threshold of conscious awareness (Libet et al., 1983; Libet, 2004). Related to the issue of determining objective indices for measuring consciousness of sensory stimuli, three main signatures have been hypothesized namely: (i) an amplification of activation and reverberation within a large network of distant brain areas (accompanied by a broad component of EEG called P300 wave); (ii) a late and distributed burst of local high-frequency activity (>30 Hz); and (iii) a massive increase in the synchronicity between distant brain areas in the beta band (13–30 Hz) (Dehaene, 2014; Dehaene et al., 2014). In recent studies combining TMS and EEG, a perturbational complexity index (PCI) has been obtained that measures cortical connectivity (Casali et al., 2013; Sarasso et al., 2014). PCI discriminated different levels of consciousness not only in healthy individuals during wakefulness, sleep, and anesthesia, but also in patients who had emerged from coma and recovered a minimal level of consciousness. In particular, inter-cortical connectivity was higher during awake and REM states and lower during slow-waves sleep, anaesthesia and minimal consciousness states (Casali et al., 2013).

## Neurobiological correlates of self and world representations: from humans to other vertebrates

**Figure 1:**
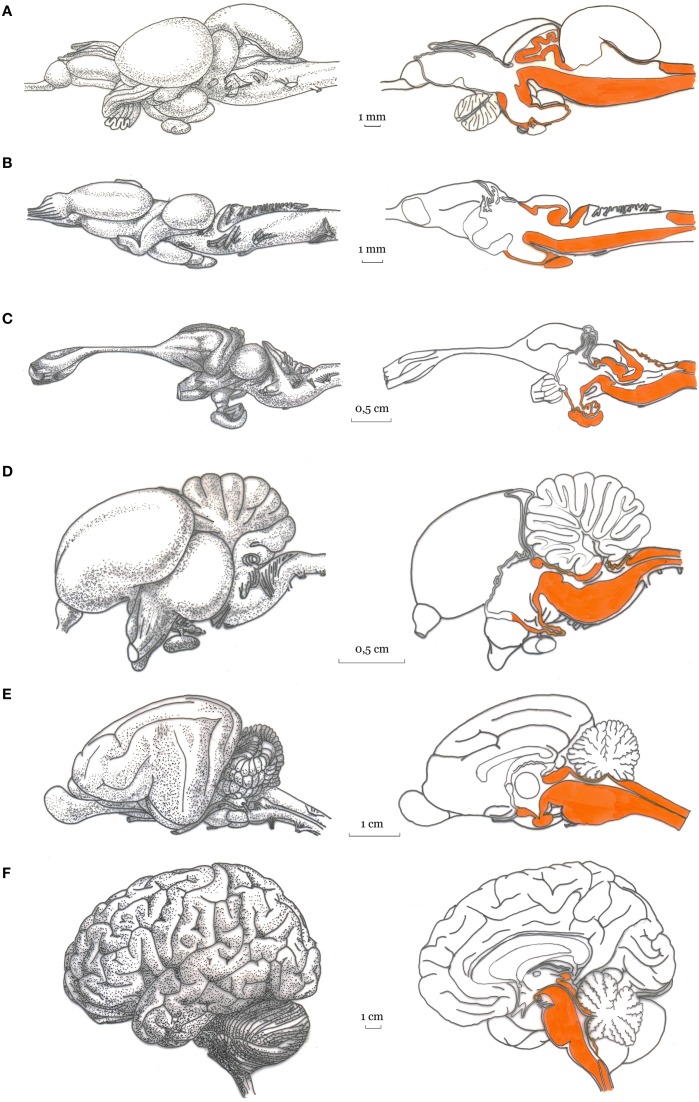
**Artistic representation of the basal and forebrain regions in the vertebrates (Image credits to Massimo Bergamasco)**. Lateral view (Left panel) and mid-sagittal view (right panel) of the brain of: **(A)** rainbow trout, Salmo gairdneri; **(B)** green frog, Rana esculenta; **(C)** tegu lizard, Tupinambis teguixin; **(D)** pigeon, Columba livia; **(E)** cat; and **(F)** human brain. For each vertebrate brain, the basal subcortical regions is highlighted (in orange) in the mid-sagittal views.

With the aim of developing our general argument, here we posit that the human brain is composed of a basal and a forebrain system (see Figure 1).

The first, hereafter referred to as basal system includes the spinal cord, the brainstem, the cerebellum, the hypothalamus, the central thalamic nuclei and the oldest portions of the telencephalon (Sarnat and Netsky, 1981; Nieuwenhuys et al., 1998; Northcutt, 2002; Striedter, 2005). The second, referred to as forebrain system, includes the evolutionarily most recent parts of the thalamus, basal ganglia and the cerebral cortex, which according to the widely known model proposed by MacLean (1990) corresponds to the triune brain (reptilian, paleomammalian, and neomammalian). Although very fortunate, the triune brain model ignores the fish brain that is instead considered in our two-systems proposal. Indeed, while the forebrain system is developed in the vertebrate terrestrial animals (although with various degrees of complexity in amphibians, reptiles, birds, and mammals), only the basal subcortical system is well developed in fishes. Here we posit that a form of primary consciousness is already present in the animals with a nervous system composed mainly by the basal subcortical system (e.g., in fishes, Damasio, 1999, 2010; Chandroo et al., 2004; Merker, 2007; Ward, 2011). Interestingly, the reappearance of consciousness in people emerging from general anesthesia is related to activity of brainstem and diencephalic structures (Långsjö et al., 2012; Mashour and Alkire, 2013a).

Studies indicate that basal subcortical structures play an important role in the representation of self and world (see for example, Panksepp, 1998a; Merker, 2007; Damasio, 2010). As mentioned above, subcortical circuitry has already reached a strong development in fishes and is present in all the other classes of vertebrates. One main function of those brain regions is to coordinate and control both the cephalic segment and the remaining parts of the body, with a variety of brainstem structures including some of the most ancient, centromedial regions like the periaqueductal gray (Bailey and Davis, 1942; Panksepp, 1998a,b) that contribute to this task. Three other midbrain and brainstem structures seem to play a critical role for the development of primary forms of consciousness namely: (i) the hypothalamus, which is primarily involved in monitoring body homeostatic (e.g., hunger, thirst, and thermoregulation) and emotional states, and in regulating and integrating primary motivational states related to goal directed behaviors; (ii) central thalamic nuclei, particularly the thalamic reticular nucleus, that are thought to be involved in maintaining primary conscious awareness (Ward, 2011); (iii) the roof of the midbrain (superior colliculus or optic tectum), which represents a multisensory integration station where representations (simulations) of a “distal world” are generated; and as already noted (iv) the periaqueductal gray (PAG) a structure of fundamental importance for the emotional-motor integration that has recently received abundant attention (Parvizi and Damasio, 2001; Merker, 2007, 2013; Panksepp and Northoff, 2009; Damasio, 2010).

The roof of the mesencephalon of vertebrates (optic tectum), together with the hypothalamus (located in the floor of the diencephalon), forms the mesodiencephalon, a structure with major integrative functions present in all vertebrates. It has to be noted that superior colliculus (and/or the optic tectum) has a special role in controlling eye movements that are probably the most complex movements present in fishes (Stein et al., 2002a,b). The PAG is a complex mesencephalic structure that surrounds the cerebral aqueduct, is intimately related to the deeper collicular layers, and is part of the emotional motor system. In mammals it receives more than half of the ascendent fibers related to pain perception (Parvizi and Damasio, 2001; Damasio, 2010), and is strongly connected with important structures of the basal subcortical system (hypothalamus, amygdala, raphe nuclei, and superior colliculus) as well as with structures of the forebrain group (insula and cingulate cortex; Vianna and Brandão, 2003). The PAG is probably one of the most ancient structures devoted to emotional integration, it coordinates an exceptional variety of emotion-related behaviors such as defensive, aggressive, reproductive, vocal, and pain-related ones, from fishes to human beings (Lovick and Adamec, 2009). The importance of PAG in emotional integration is particularly evident from the analysis of the very rare patients with global selective PAG lesions, comparable to the cats and monkeys studied by Bailey and Davis (1942). Patient GM, for example, although not affected by primarily motor disturbances, remained immobile for hours, completely dumb and lacking any motor initiative. This behavior was also in contrast with the patient's ability to understand complex phrases and execute complex orders (Esposito et al., 1999).

Anatomic, physiological and behavioral evidence suggests that the basal subcortical system may underpin a primary self-representation. Such a system, which is present in all vertebrates, is made of neural circuits based on reciprocal connections between brain stem and central thalamic nuclei with phylogenetic old cortices (Meek and Nieuwenhuys, 1998; Mueller, 2012). These connections may allow for the type of re-entrant interactions that, according to Edelman, are at the base of primary consciousness (Edelman, 1992, 2004; Edelman and Tononi, 2000). Neurophysiologic studies indicate that brainstem structures are endowed with a neural machinery adept to perform multimodal integration of sensory stimuli and create multisensory representations of the self and the body (Herrero et al., 1998; Sparks, 2002; Luque et al., 2005) and allow organisms to integrate world- (target selection), body- (action selection), and motivation- (needs) cues so as to optimize decisional processes (Merker, 2007; Broglio et al., 2011). Moreover, the superior colliculus is the only structure outside the cerebral cortex where fast oscillations in the gamma range associated to the conscious perception of stimuli have been recorded (Brecht et al., 2001; Engel and Singer, 2001; Fries, 2009). Behavioral studies indicate that in teleost fishes lesions of the tectum but not of hippocampal pallium, impair the use of egocentric strategies for spatial orientation and generates a profound disorganization of exploratory patterns (Salas et al., 2003; Burnett et al., 2004). Clinical studies indicate that anencephalic humans can exhibit minimal states of consciousness as indexed by affective interactions with other individuals and absence seizures (Shewmon et al., 1999; Merker, 2007).

The nervous system structures that we assign to the basal subcortical group seem to be capable of generating a somewhat rudimentary representation of the world and of the self in mobile animals. In particular, the superior colliculus and the periacqueductal gray may be involved in the creation of an ego-centered, primary consciousness that is crucial for generating a representation of the world (Stein et al., 2002b; Panksepp and Northoff, 2009; Damasio, 2010). It is from the interaction between subject-centered perspectives and objects that a conscious mind can appear (Damasio, 1999, 2010; Revonsuo, 2006). More specifically any conscious state implies the presence of a subject (“x”) in relationship with an object (“y”) situated in a specific space (“z”) (Merker, 2007, 2013); and for human beings also the variable time (“t”) seems to be relevant. However, there is a possibility that time is completely a construction of the brain to code for changes in the world rather than being a entity that exists in the world. For instance, schools of physics have emerged suggesting the universe itself may be timeless (Barbour, 2000). That self and world representations are linked to the basal subcortical group was posited by the famous neurosurgeon Wilder Penfield who thought that studying the cortical cerebral effects of epilepsy could provide a unique opportunity to study the neuronal substratum of consciousness (Penfield and Jasper, 1954, p. 480; Penfield, 1975). Although not widely enough recognized in consciousness studies, it is now eminently clear that affective consciousness is a property of subcortical circuits we share with the other animals (Panksepp, 1998a; Solms and Panksepp, 2012).

## Evolution and organization of the nervous system in vertebrates

All vertebrates derive from elongate worms with flat or cylindrical bodies (protochordates) living in fresh water, like the planarians, with a primitive circulatory system and the enteric tract opened at both ends (Sarnat and Netsky, 1985, 2002; Nieuwenhuys et al., 1998). Vertebrates are divided in jawless vertebrates (agnates) and jawed vertebrates (gnathosomes). The earliest vertebrates, namely the jawless fish, appeared soon after the Cambrian period, about 470 million years ago (the corresponding actual forms are Hagfishes and Lampreys). The first amphibians (ancestors of the present frogs, toads, newts, and salamanders) left water around 370 million years ago. The terrestrial life required the development of a respiratory system and limbs to permit survival and active movements. Around 300 million years ago the first reptiles appeared. They were able to move with larger efficiency on dry land. They laid eggs on dry land and developed copulatory organs allowing internal fertilization. Soon after their origin, reptiles divided into three separate lineages: the synapsidis, that diversified into mammals, the anaspides the ancestors of present-day turtles, and the diaspides that led to dinosaurs, birds as well as to other living reptiles (crocodilians, lizards, and snakes). Both birds and mammals have developed thermoregulation. In these classes a substantial amount of energy is utilized to maintain stable body temperature. This fact had other major consequences, for instance the need for infant birds and mammals to be cared for a long time after birth, a phenomenon that likely fostered social-bonding, mechanisms of early socialization and various learned behaviors. The first mammals developed during the same general time period when dinosaurs reached their evolutionary peak (around 220 million years ago), but they generally occupied nocturnal niches. For this reason mammals may have developed an efficient ability to move in the dark using sense of smell and a representation of space based on the sense of hearing (Jerison, 1973). The augmented hearing abilities of mammals allowed them to develop specific forms of communication between babies and their mother (Allman, 2000).

## World and self representations in the main classes of vertebrates

Although our ultimate aim is to define what is unique to human consciousness—(encapsulated by Endel Tulving's concept of autonoetic consciousness), we discuss in the following evolutionary perspectives about the existence of consciousness in other creatures. Although this has long been a contentious topic, we accept that consciousness comes in various forms ranging from affective value-encoding varieties (see Panksepp, 1998a) to a diversity of cognitive instances. Moreover, consciousness has an evolutionary history. Thus, the experiences of other animals deserve the respect and understanding that has not consistently been part of the scientific conversation. Thus, although briefly, we highlight here a variety of key issues.

Several studies attempted to define stringent criteria for exploring consciousness in non-human animals (Edelman et al., 2005; Seth et al., 2005; Edelman and Seth, 2009; Boly et al., 2013). Given the absence of language in non-human species, information about their ability to represent the self and the world cannot be obtained as explicit reports concerning the introspective, first-person perspective. However, the following anatomic and physiologic evidence hints at the presence of primary consciousness in non-human animals: (i) a pattern of EEG activity in the range of 20–70 Hz, typically associated to wakefulness and REM sleep (Revonsuo, 2010); (ii) thalamo-cortical activity (Edelman et al., 2011); (iii) widespread brain activation during processing of sensorial stimuli (Baars, 2002; Tononi, 2012); (iv) selective synchronization at cortical and brainstem levels of dynamically formed neural networks involved in binding sensory stimuli (Brecht et al., 2001; Crick and Koch, 2003); (v) presence of egocentric maps for localizing an individual in a given space. Although mainly found in the parietal lobe, egocentric, proto-self related maps are also found in the limbic region, the hypothalamus, and even in the PAG and in the mesencephalon (Parvizi and Damasio, 2001; Damasio, 2010). Indeed, if one considers affective states to be part of phenomenal consciousness, then it is noteworth that such systems are concentrated in subcortical regions of the brain (Panksepp, 1998a; Solms and Panksepp, 2012). It is worth noting that invertebrates, such as crayfish, also exhibit conditioned place preferences for drugs like amphetamine, cocaine, and morphine that are highly addictive in humans. This is consistent with the thesis that even some invertebrates may have affective experiences (see Huber et al., 2011). However, here we will largely consider evidence for vertebrates, with a focus on cognitive rather than affective forms of consciousness, which may go even further back in brain evolution.

### Fishes

Fishes are the largest class of vertebrates with more than 32,000 species. Most of the available knowledge about fishes comes from the study of Teleosts in which the nervous system is organized similarly to the other main classes of vertebrates. In particular, the brain of all vertebrates shares the main classes of neurotransmitters and neuromodulators and the topographical organization (with spinal cord, the rhomboencephalon—medulla oblongata, pons and cerebellum-, the mesencephalon, and the prosencephalon—diecephalon and telencephalon (Meek and Nieuwenhuys, 1998; Mueller, 2012).

In several species of Teleosts the most developed sensory organ is the eye. Around five hundred thousand fibers of the optical nerve reach the optic tectum (a retinotopically-organized structure composed of seven main layers) that integrates visual, auditory and tactile information in order to build up a body-centered representation of the environment. Moreover, the optic tectum coordinates the oculomotor circuit that is the most complex motor system present in fishes. Indeed, fish that exhibit the best discriminatory behaviors show the most developed “voluntary” eye movements, including, in some cases, individual fixation movements of the two eyes (Trevarthen, 1968, p. 89; Sagev et al., 2007; Martinez-Conde and Macknik, 2008). Moreover, neuropsychological deficits in allocentric (i.e., world-based) visual coordinates but not in ego-centric (i.e., self-based) motor coordinates have been observed in Teleost fishes after lesions of the telencephalon, while optic tectum lesions brought about deficits in egocentric, body-centered orientation, an effect also found in vertebrates (Broglio et al., 2011). It is worth noting that lesions to the medial telencephalic pallium (a structure corresponding to the amygdala) in the same species induced significant disorders in emotional behaviors (aggressive, reproductive, parental behavior, and emotional memories; Chandroo et al., 2004; Salas et al., 2006). Studies indicate that goldfish trained to use allocentric procedures based on distal visual cues can navigate directly to the reward place from previously unvisited start locations by adopting spontaneously the most direct routes. The use of appropriate trajectories without a history of previous training represents evidence for the capacity of goldfish to represent the environment independently from a body-centered reference system (Odling-Smee et al., 2012). Interestingly, allocentric spatial cognition in fish is lost following lesions to the teleost telencephalon. In contrast, body-centered orientation strategies are impaired following tectal lesions (Broglio et al., 2011).

Shoaling fishes use odor and visual cues to perform sophisticated discrimination of their preferred shoalmates suggesting individuality can be recognized (Griffiths and Ward, 2012). Dugatkin and Godin (1992) provide experimental evidence that female guppies change their preference between two males if they observed another female being courted by the less-preferred male. Also, males cichlids (*Astatotilapia burtoni*) can perform transitive inference about dominance hierarchy by observing the interactions of co-specifics with different degrees of dominance (Grosenick et al., 2007) and deciding to spend more time close to the weaker male (Bshary, 2012). Thus, several types of fishes have impressive long term memories and show complex behaviors that are reminiscent of the Machiavellian strategies of manipulation observed in more complex species (Brown et al., 2011). Studies indicate that fishes have the ability to perform self-discrimination tasks. The cichlid *Pelvicachromis taeniatus*, for example, can recognize its own odor and prefer it's over the odor of other, either familiar or unrelated fishes (Thunken et al., 2009). Moreover, fishes appear able to assess their own status and make behavioral decisions based upon this notion. It is also worth noting that the behavior of fishes merely watching the fight of conspecifics is influenced by the victor or loser of the interaction. This suggests that fishes are able to make third-party assessment relative to others (Oliveira et al., 1998). Based on the role that specific structures play in the different forms of consciousness in mammals, one may hypothesize that Teleost fishes may have a “primary cognitive consciousness” (a primary representation of the world, with objects, and the proto-self) and possibly also a “core self” (see Table 1; Griffin, 1992; Chandroo et al., 2004; Merker, 2007; Broglio et al., 2011). What is not clear is whether at least some Teleost fishes show the eye movements and motor automatisms that characterize REM sleep in mammals (Nicolau et al., 2000). Neural systems responsible for normal sleep-waking cycling are localized in the brain stem while the system related to REM sleep generation are localized in structures below the ascending reticular activating system responsible for waking arousal. Thus, while REM mechanisms may be related to a primitive form of waking arousal that provides background functions such as the integration of emotionally information, higher-brain stem structures are responsible for a more complex form of primary consciousness (Panksepp, 1998a).

**Table 1:** **Representation of the self in the main classes of vertebrates**.

	**Primary self**	**Core self**	**Self-consciousness**	**Narrative self**
Teleost fishes	+	(?)	−	−
Anphibians	+	(?)	−	−
Reptiles	+	+	−	−
Birds	+	+	+^*^	−
Mammals	+	+	+^**^	−
Homo sapiens	+	+	+	+

* *European Magpie (Pica Pica)*.

** *Chimpanzee, Orangutan, Dolphin, Elephant*.

### Amphibians

Most of our knowledge about the amphibians' (frogs, newts, and salamanders) brain comes from studies of frogs (Ten Donkelaar, 1998a; Llinás and Precht, 2012). These animals do not possess the fovea, are not able to perform eye movements but use visual information to capture preys (by catching them with the tongue) and to recognize potential predators and to escape. On the other hand, their sexual life is regulated by touch, hearing and vocalizations. The studies performed by Lettvin et al. (1959) showed that frogs are able to see only moving objects. In constrast, studies by Ingle (1973) described two visual pathways also in frogs: (i) the first (the tectal system) is involved in prey catching and in the visually-driven escape from looming targets; (ii) the second (the pretectal system) is responsible for visual guidance to avoid barriers and obstacles. The most complex structure processing visual, auditory and tactile inputs in frogs is the optic tectum that may be responsible of the representation of the world and of the proto-self. In particular, Jörg-Peter Ewert studied how frog and toads use visual information to recognize prey and predators: a small horizontal stripe (worm configuration) is interpreted as prey, a vertical stripe (antiworm configuration) is interpreted as a potential predator (Wachowitz and Ewert, 1996; Ewert, 1997). Amphibians show procedural learning phenomena (habit learning) but they do not seem to show emotional responses to stress (emotional fever and emotional tachycardia; Cabanac and Cabanac, 2004). For this reason it is held that amphibians do not show emotional responses to fear and pleasure (Cabanac et al., 2009). Yet, the organization of the amphibians nervous system, with a highly developed optic tectum and the presence of telencephalon components associated to the representation of emotions do suggest that a primary consciousness may be present in amphibians (Table 1), and as already noted, an affective form of consciousness is evident even among invertebrates when drug-reward is used as a criterion (Huber et al., 2011).

### Reptiles

The four orders of reptiles (turtles, Chelonia; tuatare, Rynchocephalia; lizards and snakes, Squamata; alligators and crocodiles, Crocodilia) together with birds and mammals, form the group of amniotes that completes their reproductive cycle outside water. The brains of reptiles generally have about double the volume as compared to amphibians, even after taking into account their body weight. Among the structures that are mainly responsible for the brain volume increase are the tectum of mensencephalon, the cerebellum and the forebrain. In diurnal reptiles the optic tectum is a structure devoted to sensory-motor integration (visual, auditory, tactile, and proprioceptive), heavily connected with the thalamus and the telencephalon. In reptiles, as well as in fish and amphibians, the tectum generates a body-centered map of the environment. The increased volume of the reptile's cerebellum is linked to the increased importance of the limbs in locomotion.

The reptile's telencephalon consists of the olfactory bulbs and the cerebral hemispheres. Each of the cerebral hemispheres contain the pallium, the striatum and the septum. The cerebral cortex of reptiles is multilayered and receives ascending projections from thalamic nuclei. One of the most significant difference between the brain of amphibians and that of reptiles is the development of a large telencephalic intraventricular protrusion: the Dorsal Ventricular Ridge (DVR). The anterior part of DVR is the telencephalon locus devoted to sensory (somatosensory, gustatory, visual, acoustic) integration and it has analogies with the mammalian neocortex. The posterior dorsal ventricular ridge (PDVR) resembles the mammalian amygdala. In reptiles the major output center of the telencephalon—the striatum—resembles the basal ganglia in mammals (Ten Donkelaar, 1998b). The existence of map-like memory representations of the environmental space and their damage following lesions to the medial cortex has been demonstrated in reptiles (Rodríguez et al., 2002).

Reptiles do exhibit “taste aversion learning” phenomena. For example, presentation of a new food associated to intra-peritoneal injections of lithium hydrochloride (a substance that produces nausea) induces long-lasting avoidance of that food not only in mammals and birds but also in reptiles suggesting that they can experience displeasure and pleasure. Similarly, physiological responses like tachycardia and fewer induced by handling can be seen in mammals and birds but also in reptiles (Cabanac, 1996, 2009; Cabanac et al., 2009). As to higher-order functions, observational learning has been found in reptiles (*Pogona vitticeps*) that proved able to open a door to obtain food reward by simply seeing a co-specifics doing the task (Kis et al., 2015). It is also worth noting that some species of reptiles (chameleon lizard, *Ctenosaura pectinata*), exhibit physiological indices of REM sleep which suggests the presence of some forms of primary consciousness (Siegel, 1999, 2008).

### Birds

Modern birds include more than 20 orders and display a great diversity of adaptation to different ecological niches. Birds possess a highly developed telencephalon. The cerebralization index (weight of the cortex/weight of the brain stem) is highest in passeriform birds (particularly in corvids) and lowest in Gallinaceae. The most developed exteroceptive sense in birds is sight. For this reason birds have very well developed tectum mesencephalic tissue—brain regions devoted to multisensory integration allowing them to build complex spatial maps of the world. The avian diencephalon is well differentiated and densely connected with the telencephalon. In particular the ventral thalamus is a relay station for visual, auditory and somatosensory information.

In birds, the telencephalon is also particularly well developed, including the paleostriatal complex and regions deriving from the DVR (neostriatum, hyperstriatum ventral, and archistriatum; structures that are homologos to the mammalian neocortex). The paleostriatal complex forms the basal part of the hemisphere and corresponds to the mammalian corpus striatum (nucleus caudatus, putamen, globus pallidus). The neostriatum is located above the paleostriatal complex and extends to the frontal, intermediate and caudal portions of each hemisphere. The neostriatum consists of two main regions namely: (i) the nucleus basalis that receives auditory information and somatosensory information coming from oral regions; (ii) the ectostriatum, that receives mainly visual information. The ventral hyperstriatum (HVC) is located on the top of the neostriatum to which is heavily connected, and is involved in visuomotor activity and learning. The archistriatum is located in the parieto-occipital pole of the hemisphere and receives afferent information from the neostriatum, the ventral hyperstriatum and the contralateral archistriatum. The archistriatum intervenes in the control of premotor and motor regions of the brain stem.

In songbirds the nucleus robustus, a structure of the archistriatum, projects directly to the nuclei involved in vocalization and respiration (the nucleus robustus together with the HVC form the “telencephalic pathway of learned song”; Nottebohm, 1993). The amygdaloid parts of the achistriatum are involved in various emotional regulations of behavior as in mammals, including aggression, fear, and sexuality. The telencephalon of birds consists of: (i) the dorsal eminentia sagittalis, a multilayered structure, involved in vision and resembling, in terms of organization, the mammalian lateral geniculate; (ii) hippocampus and area parahippocampalis, two structures involved in spatial memory and cognition (Dubbeldam, 1998).

Birds possess extraordinary learning, emotional, and cognitive capabilities (Vallortigara, 2000; Edelman et al., 2005). Birds, like mammals, have well-developed slow wave sleep and rapid eye movement sleep (Nicolau et al., 2000). The presence of REM sleep is probably associated to the ability to dream and to the presence of phenomenal consciousness (Butler and Cotterill, 2006; Revonsuo, 2006, 2010). From a perceptual point of view, chickens (Gallus Gallus forma domestica) are able to detect contours, to perform amodal completion tasks, to recognize Kaniza triangles and to perceive depth (Vallortigara, 2005). Ash-gray parrots (*Psittacus erithacus*) are able to label objects, to learn to generalize and to learn concepts (Pepperberg, 1999, 2008; Pepperberg and Lynn, 2000). Finally, the ability for mirror self-recognition seems to be present in corvids (European Magpie, Pica Pica) (Prior et al., 2008; cf. Table 1).

### Mammals

Mammals derive from a group of reptiles (terapsids) that probably adapted themselves to live in nocturnal niches. Among the distinguishing characteristics present in all mammals are: (i) the control of temperature (endothermy); (ii) the bringing up and care of the offspring; (iii) the vocal communication between the progeny and the mother; (iv) the development of the telencephalon, with the neocortex able to potentiate analytic, associative and synthetic abilities. Although the general structure of the central nervous system is similar in all mammals, relevant differences in the expansion of the cerebral cortex are found (Voogd et al., 1998).

The most primitive living mammals (e.g., the opossum) are omnivorous, live in nocturnal niches and primarily exploit the senses of smell and hearing for orienting in the world. Carnivorous are in general nocturnal predators that have developed acoustic and visual senses in addition to smell. Primates are diurnal omnivore and live on trees, have strongly developed vision, high manual dexterity and advanced social habits. All mammals are endowed with well-developed thalamo-cortical, cortico-striatal and cortical-pontine-cerebellal circuits (Seth et al., 2005). Direct cortico-motoneuronal connections that allow the execution of precise movements are highly developed in primates.

According to Jerison (1973), mammals appeared more than 200 millions year ago by occupying nocturnal niches free from reptiles. Mammals exhibit thermoregulation (endodermia), and have evolved the capacity to retain eggs internally, giving commonly to immature (altricial) offspring, except for prey species that are born precocious, ready to move soon after birth alongside mothers. In both cases, progeny are born cognitively immature, requiring prolonged maternal care. A fundamental aspect of adaptation to nocturnal niches of the earliest mammals was that their perceptual apparatus could not be based mainly on vision but had to be “re-represented” using the olfactory and auditory systems. In mammals, hearing was coordinated with vision as senses specialized for distance detection. However, the processing of auditory stimuli for the creation of spatial maps could not be developed at the peripheral level (due to the constraints of the auditory channels) but was moved to more central loci, involving several structures at both brainstem and cortical levels.

Primates, by adapting to the life on trees, have developed a second visual system which is heavily cortical (although closely coordinated with the older retino-optic tectal system; Goodale and Milner, 2004). The development of the cerebral cortex is probably responsible for a diversity of higher mental abilities that emerge developmentally, largely by learning, in mammals. The widespread presence of REM sleep is an indicator of the existence of varieties of dream-consciousness in mammals (Revonsuo, 2006, 2010). Since dream-consciousness is expressed at highest levels during the weeks and months following birth, the suggestion is made that it may serve to refine critical developmental functions. It is also noteworthy that mirror self-recognition has been demonstrated in diverse mammalian species (Vallortigara, 2000; Tannenbaum, 2009; Devue and Brédart, 2011; cf. Table 1).

From both neuroanatomical and neurophysiological perspectives, human beings have the most complex central nervous system with a very high brain-body scaling (brain weight vs. body weight) index. What is greatly increased in the human cerebral cortex is the number of inter-areal connections which is 2.75 times with respect to chimps, with number of neurons being only 1.25 higher (Deacon, 1990). Moreover, the human brain exhibits an extraordinary development of the prefrontal lobes and of a direct cortico-spinal bundle that allows the execution of fine movements of eye and hand muscles as well as of the vocal articulatory system (Jerison, 1973; Voogd et al., 1998; Striedter, 2005). Although the higher-order brain regions alone may not have the intrinsic ability to sustain consciousness (Solms and Panksepp, 2012) it is clear that the mental abilities that emerge from neocortical dynamics distinguish us as the most cognitively complex species in the world, but certainly not the only one that has consciousness.

## What is like to be a human?

It has long been held that language is what makes humans unique (Lakoff and Johnson, 1980; Pinker, 1994; Hauser et al., 2002). However, here we contend that at least two additional important abilities are at the very root of being humans. The first is development of theory of mind and the second the ability to mentally travel in time that has been considered a most sophisticated form of auto-noetic consciousness. Of course, we cannot exclude such possibilities in other animals, for detection of such abilities is critically dependent on complex language abilities. Although it is certainly possible that other animals may visually or even with other senses, imagine future possibilities, only humans seem to be able to conceive their death (Humphrey, 2011; Berlucchi, 2014; Corballis, 2014).

In any event, the evolutionary pressure to develop increasingly complex brains seems maximal at the transition from *Homo habilis* to *Homo sapiens*, possibly promoted by the adaptive value of an ever increasing complexity of social interactions. Indeed, to maximize survival in diverse environments and among diverse predatory animals, it is fundamentally important to understand the thoughts, intentions and plans of others, an ability that is encapsulated in the concept of “theory of mind,” i.e., the ability to detect and respond to the mental states of others (Goldsmith and Zimmerman, 2001; Baron-Cohen, 2011; Corballis, 2012).

Several vertebrates are able to represent spatial aspects of the world and the self as well as to represent time. However, human beings may be the only ones who developed sophisticated mental capacities for representing how one's existence is related to the thoughts and behaviors of others, in both space and time. The aspects of consciousness which allowed humans to develop the cognitive/affective dynamics of social interactions to be internalized within brain/mind experiential spaces, might parallel the massive expansion of the cerebral cortex and of the connectivity across different cerebral areas. Although the capacity for phenomenal consciousness is strongly represented already in the subcortical neural circuits we share with other animals, our vast cortical networks allow us to develop the most complex mental attitudes both toward others and the world.

These higher-order aspects of cortical functions are strongly allows humans to encode in social and environmental complexities the highly sophisticated temporal characteristics, that allow to develop mental landscapes that permit strategizing above and beyond the capacities of any other species. Indeed, humans may be the only ones who are not only aware that they live in the present moment but also along subjectively experienced time lines of the past and future. Such complex awareness of material and social worlds is inherently linked to our “autonoetic consciousness,” and allows human beings to mentally represent and become aware of their protracted existence in time and space (Wheeler et al., 1997). Thanks to this ability humans may not only reconstruct events of their past but imagine various possible futures.

In the mid 80s Endel Tulving described an amnesic patient (K.C.) who was no longer able to memorize and remember events of his past and, more relevant here, to imagine his future. The loss of the subjective experience of time (autonoetic consciousness, self-knowing), in spite of preserved semantic memory (noetic consciousness), brought KC to live in a permanent present with no subjective sense of time but completely spared cognitive knowledge about physical time, its units, its structure, and its measurement by clocks and calendars. Using Tulving's (2002b, p. 317) words, the patient exhibited “a dissociation between knowing time and experiencing time, a dissociation that parallels the one between knowing the facts of the world and remembering past experiences.” Subsequent neuroimaging studies showed that the cerebral structures that processed the remembering of the past and imaging of the future (mental time travel) largely overlap and include: (i) medial prefrontal regions; (ii) medial (precuneus) and lateral parietal cortex; (iii) medial temporal lobe; and (iv) lateral temporal lobe (Schacter et al., 2007). To mentally travel in time it is necessary to develop: (i) a sense of subjective time; (ii) the ability to be aware of subjective time (autonoesis). Moreover, a traveler has to be a “special self” able to imagine its existence in time (Tulving, 2002a). Mental time-traveling abilities are not present in toddlers and start to develop only 6 months, after the ability for mirror self-recognition has emerged. Only around 23 months, toddlers start to utilize temporal markers for the past and are able to foresee future activities on the basis of past experiences (Howe et al., 2003). Thus, autonoetic consciousness, that may be well-developed around age 3, appears necessary although not sufficient for the development of episodic memory (Wheeler et al., 1997; Buckner and Carroll, 2007).

At any rate, the awareness of self in time constitutes the typical existential dimension of life that ultimately allows human beings to become aware that death is unavoidable (Heidegger, 1972; Fabbro and Crescentini, 2014). Although some capacity for episodic, self-referential memories has been demonstrated in other species, such as certain species of birds (Clayton et al., 2003), the mental dimension of mortality, and all that implies, does not seem to be present in any other living species (Suddendorf and Busby, 2003) and it might have been crucial for human cultural evolution (Tulving, 2002b). The construction and conservation of tools (lithic) may be possible only in the light of their possible future utilization (Fabbro and Tomasino, 2012; Fabbro and Crescentini, 2014). Moreover, verbal structures indicating past and future cannot develop without a subjective awareness of time. In conclusion, the narrative self, one of the most advanced forms of conscious representation of the self and of the world, can probably develop only in individuals who have developed a subjective consciousness of time and a sufficient capacity for symbolic/verbal communication. Therefore, mental time traveling may also have to do with developing unique skills that distinguish human beings from other animal species like the linguistic and technological skills underlying human cultures (Corballis, 2002, 2012; Lieberman, 2006).

## Conclusions

All vertebrates are probably sensory-phenomenally and affectively conscious, as controlled by circadian cycles and various states of being. Dynamic transitions across different states of consciousness (e.g., from slow-wave sleep to wakefulness) are found in all vertebrates. Crucially, the range of transitions is maximal in humans in whom behavioral and neural changes linked to sleep, dreaming, lucid dreaming, hypnotic trance, out-of-body experiences, assumption of substances are described (Fabbro, 2010; Baars, 2013). Interestingly, dance and music, that are used in numerous cultures within medical and religious contexts, favor the transition from ordinary to altered states of consciousness (for overviews, see Maloch and Trevarthen, 2009). Thus, in addition to language, theory of mind and mental time traveling, the varieties of higher-order states of consciousness (Lewis-Williams and Clottes, 1998; Lewis-Williams, 2002) may have driven culture and ultimately made humans unique.

### Conflict of interest statement

The authors declare that the research was conducted in the absence of any commercial or financial relationships that could be construed as a potential conflict of interest.
